# Bladder and Bowel Dysfunction Network: Improving the Management of Pediatric Bladder and Bowel Dysfunction

**DOI:** 10.1097/pq9.0000000000000383

**Published:** 2021-03-10

**Authors:** Martha Pokarowski, Mandy Rickard, Ronik Kanani, Niraj Mistry, Megan Saunders, Rebecca Rockman, Jonathan Sam, Abby Varghese, Jessica Malach, Ivor Margolis, Amani Roushdi, Leo Levin, Manbir Singh, Roberto Iglesias Lopes, Walid A. Farhat, Martin A. Koyle, Joana Dos Santos

**Affiliations:** From the * Division of Urology, The Hospital for Sick Children, Toronto, Ontario, Canada; † Department of Pediatrics, North York General Hospital, North York, Ontario, Canada; ‡ Department of Paediatrics, The Hospital for Sick Children, Toronto, Ontario, Canada; § Faculty of Medicine, University of Toronto, Toronto, Ontario, Canada; ¶ Department of Pediatrics, Oakville Trafalgar Memorial Hospital, Oakville, Ontario, Canada; ∥ Department of Pediatrics, Markham Stouffville Hospital, Markham, Ontario, Canada; ** Department of Pediatrics, William Osler Health Centre-Brampton Civic Hospital, Brampton, Ontario, Canada; †† Faculty of Health Sciences, McMaster University, Hamilton, Ontario, Canada

## Abstract

Lower urinary tract symptoms with constipation characterize bladder and bowel dysfunction (BBD). Due to high referral volumes to hospital pediatric urology clinics and time-consuming appointments, wait times are prolonged. Initial management consists of behavioral modification strategies that could be accomplished by community pediatricians. We aimed to create a network of community pediatricians trained in BBD (BBDN) management and assess its impact on care.

**Methods:**

We distributed a survey to pediatricians, and those interested attended training consisting of lectures and clinical shadowing. Patients referred to a hospital pediatric urology clinic were triaged to the BBDN and completed the dysfunctional voiding symptom score and satisfaction surveys at baseline and follow-up. The Bristol stool chart was used to assess constipation. Results were compared between BBDN and hospital clinic patients.

**Results:**

Surveyed pediatricians (n = 100) most commonly managed BBD with PEG3350 and dietary changes and were less likely to recommend bladder retraining strategies. Baseline characteristics were similar in BBDN (n = 100) and hospital clinic patients (n = 23). Both groups had similar improvements in dysfunctional voiding symptom score from baseline to follow-up (10.1 ± 4.2 to 5.6 ± 3.3, *P* = 0.01, versus 10.1 ± 4.2 to 7.8 ± 4.5, *P* = 0.02). BBDN patients waited less time for their follow-up visit with 56 (28–70) days versus 94.5 (85–109) days for hospital clinic patients (*P* < 0.001). Both groups demonstrated high familial satisfaction.

**Conclusions:**

Community pediatricians may require more knowledge of management strategies for BBD. Our pilot study demonstrates that implementing a BBDN is feasible, results in shorter wait times, and similar improvement in symptoms and patient satisfaction than a hospital pediatric urology clinic.

## Abstract 2

Supplemental Digital Content is available in the text.

## INTRODUCTION

Bladder and bowel dysfunction (BBD) is the most common reason for referral to pediatric urology clinics and represents up to 40% of pediatric urology consults.^1,2^ BBD is often considered an umbrella term used to describe lower urinary tract symptoms (LUTS) with or without constipation.^3^ Due to the close anatomical proximity of the bladder and bowel, it is a known concern that their functioning is interrelated, and abnormalities found within 1 system will influence the other.^4–7^ This fact may explain why constipation is reported in more than 50% of children seen in pediatric urology clinics for LUTS, despite a childhood constipation prevalence of approximately 30%.^8^

The fundamentals of BBD management include bladder retraining strategies, timed and double voiding, adequate fluid intake, and constipation management. Most children demonstrate improvement, albeit with reinforcement and reassurance.^9,10^ Successful implementation of these strategies, combined with simultaneously aggressive constipation management, may take several months, and as a result, it is often undertreated by primary care providers.^8^ Persistent symptoms of BBD, such as incontinence, constipation, and/or urinary tract infections (UTIs), prompt referrals^11^ to urology clinics resulting in high volumes, lengthy wait times, and overinvestigation.

Herein, we present the results of a survey circulated to community pediatricians to determine their understanding of BBDs pathogenesis and employ management strategies to identify education gaps. We also describe the implementation of a pilot project, the BBD network (BBDN), consisting of a group of community pediatricians who received training in BBD management to provide supported initial and ongoing management of children with BBD referred from an academic pediatric urology clinic. We hypothesized that successful implementation of a BBDN would result in improved wait times for initial and follow-up visits, demonstrate comparable improvement in symptom scores, and show similar overall familial satisfaction with a reduction of BBD patients seen in a hospital urology clinic.

## METHODS

### Population and Process

Following approval by our institutional Quality Improvement committee, an online survey was distributed to community pediatricians via a medical association electronic newsletter from March to June 2016, questioning familiarity with and/or understanding of BBD, preferred management strategies, thresholds for specialist referrals, and referral reasons. Survey questions and responses can be reviewed in **Supplemental Digital Content 1,** which describes results of an online survey distributed to pediatricians regarding their experiences with BBD management, http://links.lww.com/PQ9/A240.

The BBDN, a pilot initiative, initially consisted of 7 pediatricians who expressed an interest in participation and underwent training at a quaternary care hospital for 1 day. The educational session comprised content lectures provided by pediatric urology faculty and clinical shadowing in the hospital BBD clinic. The session aimed to standardize the pediatric BBD treatment approach and provide information about treatment options and alternatives. Additionally, the pediatric urology quaternary care hospital team provided ongoing clinical support to the BBDN pediatricians. Therefore, the pediatric urology team was readily available to discuss specific cases with community pediatricians. Patients who need to be seen by a urologist are referred back to the hospital without further delays.

**Fig. 1.**
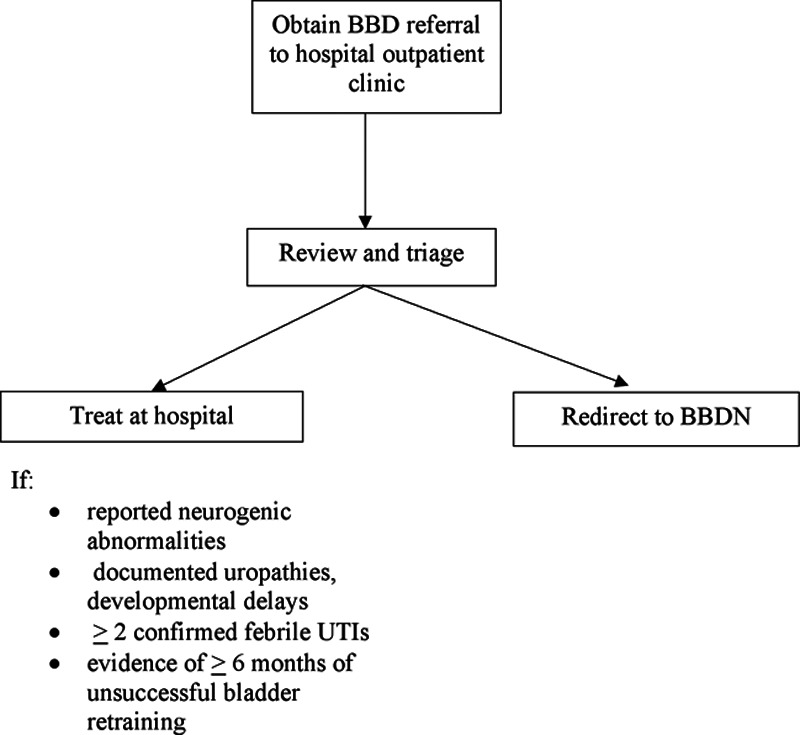
BBD referral flow diagram.

We reviewed patients referred to the pediatric urology clinic for symptoms consistent with BBD. We included children 6–17 years of age diagnosed with BBD based on clinical findings (functional voiding symptoms with or without constipation). Children with reported neurogenic abnormalities, documented uropathies, developmental delays, >2 confirmed febrile UTIs, or evidence of older than 6 months of unsuccessful bladder retraining were excluded from referral to the BBDN. Rather, they were seen in the hospital clinic. Of the appropriate referrals, a sample of these patients was selected for re-referral to the BBDN (see Fig. 1). Patients referred to our institution were re-referred to a BBDN community site closest to their area of residence. Patients were seen at the hospital clinic if it was determined that this was the closest location. We aimed to keep the level of complexity of patients seen at each community site relatively homogenous.

### Outcome Measures

An objective assessment of symptom severity at baseline and follow-up visits was measured with the dysfunctional voiding symptom score (DVSS) questionnaire and the Bristol stool chart (BSC). The DVSS quantifies the severity of abnormal voiding behaviors in children^12^ with a 10-item tool identifying the presence of voiding-related symptoms and life stressors. Scores range from 0 to 25, where a higher score indicates more severe voiding dysfunction. The BSC measures stool consistency and shows excellent agreement compared to the parental report in assessing the prevalence of functional constipation based on Rome III criteria in infants and toddlers.^13^ Scores range from 1 to 7, where lower scores suggest constipation and high scores are suggestive of diarrhea.^14^ Constipation was diagnosed when children or caregivers selected stool types less than type 4, any bowel movements less frequent than daily, or reported encopresis. Familial satisfaction ratings were measured using a 10-point Likert scale. As a proxy for satisfaction, BBDN patients/families were asked to rate how likely they were to recommend the BBDN to friends and family members on a 10-point Likert scale (see **Supplemental Digital Content 2,** which describes BBDN patient/family questionnaires completed at baseline and follow-up visits, http://links.lww.com/PQ9/A241).

### Data Analysis

We computed frequencies and percentages for the collected survey responses. Open-ended responses to one survey question were grouped into related categories by a single reviewer. Descriptive statistics were used to evaluate patient age, sex, and clinic location. Chi-square analyses were conducted to examine age group and sex differences between hospital clinic and BBDN patients. We used the Mann–Whitney U test/Wilcoxon rank-sum test to determine differences in the distribution of DVSS scores, BSC scores, wait times, and patient/familial satisfaction ratings.

The Wilcoxon signed-rank test was used to examine the presence of statistically significant differences in the distribution of DVSS scores and wait times from baseline visits to follow-up visits for hospital clinic patients and BBDN patients. We conducted all statistical analyses using STATA (Version 14.0), and a two-sided *P* value of <0.05 was considered statistically significant.

## RESULTS

### Pediatrician Survey

The online survey was distributed to 1,000 community pediatric physicians, and 103 (10%) responded. More than half (57%) of respondents stated that they diagnose BBD 1-4 times per month, and 13% reported >10 diagnoses per month. Approximately, 25% reported referring to a pediatric urologist or gastroenterologist for BBD 1–4 times monthly, with the most common reasons for referral to a subspecialist being red flags (ie, neuromuscular, spinal, or developmental concerns, confirmed febrile UTIs; 74%), a lack of response to treatment (62%), and parental request (58%). More than half (56%) classified a lack of response to BBD treatment as no symptom improvement within 3 months of initiation of therapies. Pediatricians appeared to be more comfortable managing bowel-related symptoms, as the most common treatment strategies reported were initiation of stool softeners (99%) and suggestions for dietary changes (91%). Voiding-related symptoms were not treated as extensively as bladder retraining strategies, increased fluid intake, voiding diaries, and bedwetting alarms were recommended less often than bowel management (79%, 56%, 48%, and 28%, respectively).

When asked to define the failure of BBD treatment, most (65%) pediatricians referred to persistent symptoms at varying timelines, and 24% mentioned poor compliance as a critical contributor.

### Bladder and Bowel Dysfunction Network

We analyzed data from the first 123 patients recruited from January 2016 to July 2017. Of these, the BBDN treated 100 (81%), and 23 (19%) were hospital clinic patients. The majority of patients were males (61% versus 50%; *P* = 0.35) at both hospital and BBDN sites. The age at presentation was similar [126 mo (interquartile range [IQR] 108–150) for hospital clinic patients versus 90 mo (IQR 54-132) (*P* = 0.06) for BBDN patients]. Patient demographics are summarized in Table 1.

**Table 1.** BBDN Pilot Initiative Patient Demographics

	Hospital, n (%)	BBDN, n (%)	*P*
No. patients	23 (19)	100 (81)	—
		Site A 11 (11)	
		Site B 21 (21)	
		Site C 15 (15)	
		Site D 9 (9)	
		Site E 2 (2)	
		Site F 17 (17)	
		Site G 25 (25)	
No. males	14 (61)	50 (50)	0.35
Age, mo, median (IQR)	126 (108–150)	90 (54–132)	0.06

**Fig. 2.**
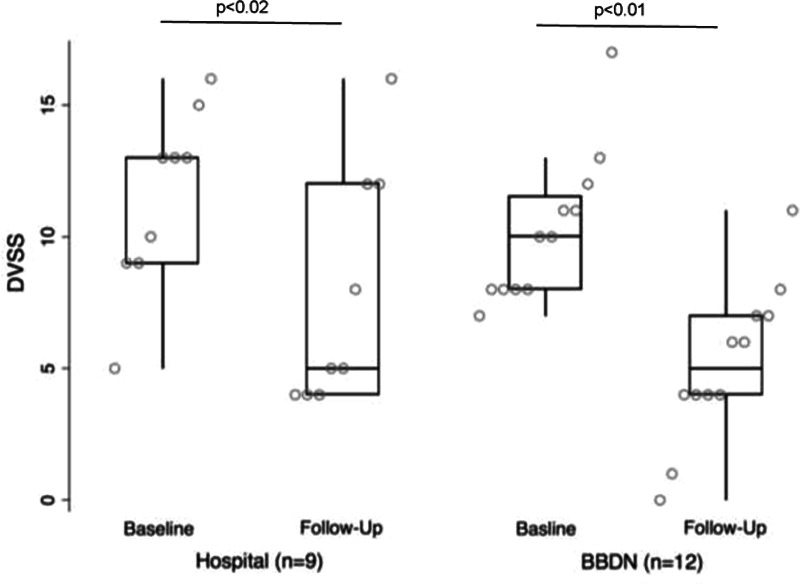
Pairwise comparisons of the median (IQR) DVSS scores for BBDN and hospital patients at baseline and follow-up visits.

**Fig. 3.**
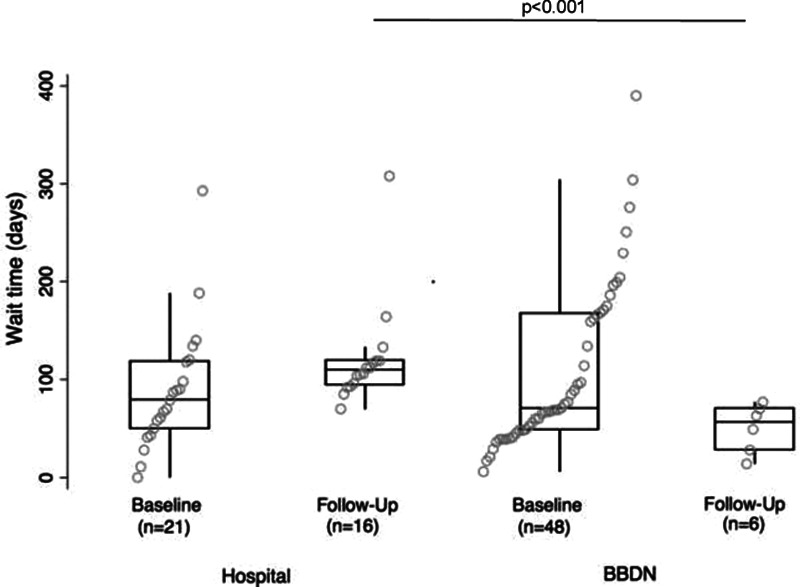
Median (IQR) wait times for BBDN and hospital patients at baseline and follow-up visits.

Outcome measures are presented in Table 2 for baseline and follow-up visits. As hypothesized, the BBDN resulted in improved wait times for initial and follow-up visits and demonstrated comparable improvement in symptom scores and overall familial satisfaction between BBD patients seen in the community and the hospital. At the initial visit, symptom severity was similar for hospital and BBDN patients with similar DVSS and BSC scores. DVSS scores significantly improved at follow-up among hospital (7.8 ± 1.5; *P* = 0.02) and BBDN patients (5.2 ± 0.9; *P* = 0.01) (Fig. 2). Stool consistency was comparable between both groups at follow-up (*P* = 0.15). Wait times for initial assessment were comparable for the hospital and BBDN patients but were shorter among BBDN patients than hospital clinic patients at follow-up visits [56 d (IQR 28–70) versus 109 d (IQR 94.5–119), respectively, *P* < 0.001] (Fig. 3). The overall familial satisfaction rating remained high among families from initial to follow-up visits with a median rating of 10 (IQR 9–10) for both hospital and BBDN patients (*P* = 0.5 and *P* = 0.76 at initial and follow-up visits, respectively).

**Table 2.** DVSS Scores, BSC Scores, Wait Time, and Satisfaction Ratings for Hospital and BBDN Patients

	Hospital, N = 23	BBDN, N = 100	*P*
Baseline			
DVSS score			
Mean (SD)	10.4 (4.3)	10.1 (4.1)	0.73
Median (IQR)	10 (8–13)	10 (8–13)	
Range	1, 19	0, 19	
Missing, n (%)	2 (9)	6 (6)	
BSC score			
Mean (SD)	2.8 (1.1)	2.9 (1.3)	0.83
Median (IQR)	3 (2–3)	3 (2–4)	
Range	1, 5	1, 7	
Missing, n (%)	5 (22)	19 (19)	
Wait time, d			
Mean (SD)	88.8 (64.9)	108.4 (84.4)	0.70
Median (IQR)	79 (50–118)	70 (48–167)	
Range	0, 293	6, 390	
Missing, n (%)	2 (9)	52 (52)	
Satisfaction rating			
Mean (SD)	9.4 (0.9)	9.4 (1.4)	0.50
Median (IQR)	10 (9–10)	10 (9–10)	
Range	7, 10	2, 10	
Missing, n (%)	6 (26)	29 (29)	
Follow-up			
DVSS score			
Mean (SD)	7.8 (4.5)	5.6 (3.3)	0.31
Median (IQR)	5 (4–12)	6 (4–7)	
Range	4, 16	0, 11	
Missing, n (%)	14 (61)	87 (87)	
BSC score			
Mean (SD)	3.1 (0.4)	3.8 (1.2)	0.15
Median (IQR)	3 (3)	3 (3–4)	
Range	3, 4	3, 6	
Missing, n (%)	16 (70)	89 (89)	
Wait time, d			
Mean (SD)	120.9 (54.2)	50.2 (24.8)	<0.001
Median (IQR)	109 (94.5–119)	56 (28–70)	
Range	70, 308	14, 70	
Missing, n (%)	7 (30)	94 (94)	
Satisfaction rating			
Mean (SD)	9.7 (0.5)	9.6 (0.7)	0.76
Median (IQR)	10 (9–10)	10 (9–10)	
Range	9, 10	8, 10	
Missing, n (%)	16 (69)	88 (88)	

## DISCUSSION

The foundation of BBD management consists of behavior modification strategies in the form of bladder retraining and constipation treatment, which ideally should be initiated by primary care providers in the community. Urotherapy is the gold standard of BBD management and is successful when applied accurately and consistently, using many different modalities of delivery.^10,15–17^ Although most children demonstrate improvement after implementing and maintaining compliance with these strategies, some do not. These are the children that may require assessment by specialist providers and more intensive investigations and management options.^9,18^ Our survey demonstrated that most pediatricians in our community were very adept at recognizing and managing constipation; however, the management of urinary symptoms and the recommendation of bladder retraining and increasing fluid intake were not commonly carried out. Also, most referrals are initiated within 3 months of the initial consultation, which generally does not allow enough time for the adequate implementation of management strategies.

Typically, urinary symptoms are resolved before constipation using a conservative treatment, as chronic constipation requires several months of management and daily bowel movements to be considered successful.^19,20^ Accordingly, the International Children’s Continence Society, which defines long-term treatment success of LUTS and associated comorbidities as the absence of relapse following the interruption of treatment after 6 months, management strategies should be implemented for at least 6 months to be considered successful or unsuccessful.^19^

Because of its inception, the BBDN has allowed a busy academic pediatric urology clinic to allocate BBD patients to community pediatricians, resulting in similar outcomes and reduced hospital volumes. Upon completing the present study, referral to the BBDN has continued, sites have expanded, and patients triaged to the BBDN earlier. After demonstrating initial success as described herein, patients referred to the hospital clinic are now triaged by pediatric urology nurse practitioners and re-referred to the BBDN directly based on the geographic location of patients and sites with no assessment by hospital-based providers, including urologists, medical urologists, and pediatric nurse practitioners. In fact, since study completion, 60% of BBD referrals have been redirected to the BBDN with the remainder being seen in the hospital urology clinic. The main reasons for the hospital clinic’s assessment were known “red flags” as documented on the referral, patients known to be more complex, or those already followed by other clinics at the hospital.

Moreover, time to initial assessment of BBD patients in the hospital clinic has increased to approximately 6 months, while BBDN continues to accommodate initial assessments within 6–8 weeks. Because we noted no difference in familial satisfaction between patients treated at the hospital than those referred to the BBDN, we are reassured that families’ expectations of treatment are being met in both settings. This observation is important as satisfaction ratings are associated with better compliance to treatment and improved health outcomes.^21^

Comparable symptom improvement among BBD patients treated in the hospital and those treated through the BBDN demonstrates that community providers can manage BBD with similar outcomes after undergoing appropriate training. The BBDN is one of the multiple collaborative networks implemented to improve the quality of care administered to community patients.^22^ The Ontario Pediatric Diabetes Network demonstrated lower emergency department visits and hospitalizations after establishing 35 specialized pediatric diabetes programs across Ontario.^23^ Another such network emphasizing in pediatric inflammatory bowel disease management demonstrated significant increases in the proportion of patients in remission at follow-up.^24^

Symptom improvement that is similar in both settings is reassuring. Although BBD may not be a life-threatening or emergent medical problem, the association of BBD with recurrent urinary tract infections and renal scarring in toilet-trained children is well documented.^9,25^ Additionally, BBD harms the quality of life and is considered a significant life stressor for both children and their caregivers.^26–28^ Fortunately, improvement in BBD symptoms results in improved quality of life. Implementation of the BBDN has allowed a more timely assessment and management of these children than offered in a traditional pediatric urology clinic. This finding translates into improved BBD symptoms and results in improved quality of life for these patients and their caregivers sooner than would otherwise be possible.

Although a novel concept and, to our knowledge, the first study describing the implementation of a network for this common childhood problem, our study is not without its limitations. Given the low response rate for our administered survey, we were only able to capture the perspectives of a small sample of pediatricians, which compromises the generalizability of physician attitudes towards BBD treatment. Furthermore, as our study was based on pilot data of the BBDN and involved a small sample size with no power calculation, caution must be taken when comparing our 2 study groups. Due to poor patient attendance observed for additional follow-up visits, we could not carry out additional longer-term follow-up analysis, which is considered necessary to determine the real impact of urotherapy. One may speculate that this could be due to improvement in symptoms and patients’ belief that no follow-up was required as previously described^29,30^; however, aside from contacting families about symptom status, this factor remains unknown. Although there were no detectable differences in outcomes between patients treated at the participating BBDN sites, we acknowledge that the pediatricians involved in their care may experience different comfort levels given their experience treating BBDN cases. With an influx of BBD patients as part of the network, this may lead to possible missed or wrong diagnoses and inappropriate treatment. Finally, the present study took place in a single-payer healthcare system at a centralized children’s hospital with a catchment area consisting of over 5 million people. All referrals for children in this area are centered in this institution, and include many from outside. As a result, the collaborative network’s implementation may not be required or feasible for locations with multiple children’s hospitals and smaller catchment areas or tiered healthcare systems.

We also acknowledge that the follow-up duration for both groups may be considered short. However, this is in keeping with other studies based on similar populations.^10,18^ Evidence of symptom improvement for both groups, despite 1 group having a shorter follow-up time, is reassuring as both groups appear to be receiving comparable care. Community pediatricians can provide closer initial support for children with BBD (which is not feasible in a large quaternary hospital) with good clinical outcomes comparable to children treated at the hospital. Close monitoring at the beginning of urotherapy offers an opportunity to adjust and individualize treatment plans, correct misunderstandings, and motivate compliance.

Despite these limitations, our study has several strengths. We prospectively examined the quality of care administered through an innovative network to treat a common pediatric problem in a traditional hospital and community setting. The implementation of the BBDN has resulted in decreased hospital clinic volumes for BBD, shorter wait times for patients, consistent follow-up visits at faster intervals than previously possible with comparable outcomes as care provided at a quaternary pediatric center. Moreover, sites of the BBDN are located in several geographic locations around the city, allowing patients to be triaged to places closer to their homes, eliminating the need for travel to the downtown core and the associated expenses. Due to the initial success described with this initiative to date, the future direction of the BBDN includes improving sites for pediatricians to provide care (ie, biofeedback and pelvic floor physiotherapists in locum) and recruiting other primary care providers such as nurse practitioners, social workers, and psychologists to the BBDN with ongoing monitoring of familial satisfaction. We plan to improve wait times and symptom improvement between all sites and expand the BBDN model to other common pediatric urology conditions that are also managed conservatively. The vast majority of these cases are phimosis, mild antenatal hydronephrosis, vesicoureteral reflux, and recurrent urinary tract infection.

## CONCLUDING SUMMARY

There appear to be barriers to BBD management by community pediatricians, who are often the entry point into the healthcare system for this population. However, additional education provided by experienced clinicians may improve these learning gaps and allow therapy initiation by these providers in their primary care setting. Given our preliminary results, the BBDN has demonstrated decreased wait times while maintaining comparable patient satisfaction and symptom improvement as patients treated in hospital without compromising care. Collaborative network models such as the BBDN could be considered in other institutions or subspecialty areas.

## DISCLOSURE

The authors have no financial interest to declare in relation to the content of this article.

## Supplementary Material


