# Supplementary material for: Bladder and Bowel Dysfunction Network: Improving the Management of Pediatric Bladder and Bowel Dysfunction
Source: Pediatr Qual Saf. 2021 Mar 10;6(2):e383. doi: 10.1097/pq9.0000000000000383 (PMC7952106; doi:10.1097/pq9.0000000000000383)
Supplement: Supplementary file 1 [file pqs-6-e383-s001.pdf]

Supplementary Data Content 1. Results of an online survey distributed to pediatricians regarding their experiences with BBD management.

		N = 100	
		N	%
Practice Location	Office-based	47	47
	Hospital	13	13
	Combination	40	40
	Missing	0	
Monthly BBD diagnoses	0	4	4
	1-4	56	57
	5-9	26	26
	10+	13	13
	Missing	1	
Monthly consultations received from other physicians for symptoms consistent with BBD	0	24	24
	1-4	53	54
	5-9	15	15
	10+	7	7
	Missing	1	
Monthly BBD referrals to pediatric urologists	0	75	76
	1-4	24	24
	5-9	0	0
	10+	0	0
	Missing	1	
Strategies used for BBD management	PEG 3350 (Lax-a-day or Restoralax)	97	99
	Dietary changes	89	91
	Bladder retraining[1]	77	79
	Increase total fluid intake	55	56
	Voiding diary	47	48
	Bedwetting alarms	27	28
	Other	18	18
	Missing	2	
Duration of BBD treatment before determining the treatment ineffective and consideration of referral to subspecialist	>1 week	1	1
	>1 month	13	13
	>3 months	42	43
	>6 months	30	31
	Other	12	12
	Missing	2	

Reasons for BBD referral to subspecialists	Red flags[2]	73	74
	Lack of response to treatment	61	62
	Parental request	57	58
	Uncertain diagnosis	35	36
	Lack of time to manage BBD	6	6
	Other	12	12
	Missing	2	

[1]Q2hr-timed voiding, double voiding, toilet posture teaching

[2]neuromuscular, spinal or developmental red flags, ≥ 2 febrile UTIs with positive cultures, and evidence of ≥ 6 months of unsuccessful voiding retraining

Abbreviations:

BBD – Bladder and Bowel Dysfunction

PEG 3350 – Polyethylene Glycol 3350
