# Supplementary material for: Bladder and Bowel Dysfunction Network: Improving the Management of Pediatric Bladder and Bowel Dysfunction
Source: Pediatr Qual Saf. 2021 Mar 10;6(2):e383. doi: 10.1097/pq9.0000000000000383 (PMC7952106; doi:10.1097/pq9.0000000000000383)
Supplement: Supplementary file 2 [file pqs-6-e383-s002.pdf]

Supplementary Data Content 2. BBDN patient/family questionnaires completed at initial and follow-up visits.

Bladder and Bowel Dysfunction Network
Please take a few minutes to fill out this survey on the quality of services you received at the BBDN and on your/your child's bladder and/or bowel symptoms. Your feedback will help us improve care for children with children with bladder and bowel dysfunction. This survey is **completely anonymous** and **voluntary**. Your answers will be kept confidential. Your/ your child's care will not be affected by your decision on participating.

Demographic Information

Site where you/your child was seen (please circle):

1. *Hospital*
2. *Site A*
3. *Site B*
4. *Site C*
5. *Site D*
6. *Site E*
7. *Site F*
8. *Site G*

Date (MM/DD/YYYY):__/__/____

For each of the following, check ✓ the response with the answer that best suits you.

What is your/your child's age bracket?	**4-10 Years**	**11-15 Years**	**16-18 Years**

What is your/your child's gender?	**Male**	**Female**

Is this your first experience at the Pediatric Elimination Network?	**Yes**	**No**

	**Second visit**	**Third visit**	**N/A**

If this is NOT your first experience at the Pediatric Elimination Network, is this your:			

Coordinating Your Care

For each of the following, check ✓ the response with the answer that best suits you.

	Strongl y Agree	Agree	Neither	Disagree	Strongly Disagree	N/A
The referral process to arrange my first visit was smooth.						
The scheduling process to arrange today's visit was smooth.						
During my visit, my appointment occurred on time.						
During my visit, I experienced long waits.						
How might we improve coordination of care at the Pediatric Elimination Network?						

Feeling Prepared

For each of the following, check ✓ the response with the answer that best suits you.

	Strongl y Agree	Agree	Neither	Disagree	Strongly Disagree	N/A
Before my visit today, I had a strong sense of what to expect.						
I was well prepared for my visit today.						
How might we help you to feel prepared for your visits to the Pediatric Elimination Network?						

Information and Education

For each of the following, check ✓ the response with the answer that best suits you.

	Strongl y Agree	Agree	Neither	Disagree	Strongly Disagree	N/A
The staff answered my questions and/or those of my child appropriately.						
The staff explained the next steps for care in a meaningful way.						
The staff gave me relevant information about mine/my child's experience that was easy to understand.						
My child and/or I were included in the decisions about care.						
The staff was sensitive to my cultural background in meeting my need for information.						
The staff explained the outcome of the clinic visit, or discharge in a caring manner.						
Is there anything we could do to improve the information or education we gave to your family to prepare for yours/your child's experience (clinic visit, discharge)?						

Connecting with the Pediatric Elimination Network

For each of the following, check ✓ the response with the answer that best suits you.

	Strongl y Agree	Agree	Neither	Disagree	Strongly Disagree	N/A
It's easy to contact the Pediatric Elimination Network staff if I have questions before/between visits.						

How might we better support you before and between your visits?	

Bladder and bowel relevant information

DVSS reviewed and answered by patient and parents

How often do you/does your child have a bowel movement?	≥ 1x/day	every other day	≥3 days

How often do you/does your child have poop accidents in the underwear?	≥ Daily	≥1x/week	≥1x/month	Rarely	Never

How does your/your child's poop looks like most of the days? (According to Bristol Stool Chart)

How much fluids do you/does your child drink daily?	≤3 cups	4-6 cups	>6 cups	Unknown /unsure

How often do you/does your child eat fruits and vegetables?	≥2x/daily	1x/day-≥3x/week	1-2x/ week	Rarely	Never	Unknown

Are you/is your child taking any stool softeners?	Yes	No

Which stool softener are you/is your child taking? Please check **ALL** that apply.	**PEG3350**	**Pico-salax**	**Bisacodyl**	**Fiber pills**	**Other (write below):**	**Unknown**	**N/A**

How often are you/is your child taking stool softeners?	≥1x/day	≥5x/week	**3-4x/week**	**Irregularly/ as needed**	**Unknown/ unsure**	**N/A**

For how long are you/is your child taking stool softeners?	**<3 months**	**3-6 months**	**>6-12 months**	**>12 months**	**Unknown/unsure**	**N/A**

How often do you/does your child pee during the day?	≤3x/day	**4-8x/day**	**>8x/day**	**Unknown/unsure**	**N/A**

How often do you/does your child have pee accidents in the underwear?	≥ Daily	≥1x/week	≥1x/month	**Rarely**	**Never**

What type of pee accidents do you/does your child have? Please check ALL THAT apply	**Daytime accidents**	**Bedwetting (never been dry at night since toilet trained)**	New onset bedwetting (after being dry ≥6 months)	Episodes of longstanding holding of urine(≥12 hours)	**N/A**

Which voiding symptoms do you/does your child have? Please check ALL THAT apply	Urgency to pee	Pees more often than normal (frequency)	Difficulty to start urination (hesitancy)	Pain/burning during urination (dysuria)	Dribbling	N/A

Did you/your child have any previous episodes of **confirmed** urinary tract infection (fever and/or voiding symptoms > 48h **AND** positive midstream/catheter urine culture)	Yes	No	Unknown/unsure	N/A

If YES, how many CONFIRMED urinary tract infections did you/your child have?	1	2-4	>4	Unknown/unsure	N/A

Overall Patient Experience

On a scale of 0-10, how likely is it that you would recommend our BBDN to a friend or family member?

0	1	2	3	4	5	6	7	8	9	10
○	○	○	○	○	○	○	○	○	○	○

Extremely Unlikely ⟷ **Extremely Likely**

Thank you for taking the time to complete this survey, your input is valuable to us. The survey can be returned to the secure collection box located in the area and identified with the label "Patient Experience Drop Box" or directly to your Pediatric Elimination Network health care provider.
